# Angiotensin II mediates the high-glucose-induced endothelial-to-mesenchymal transition in human aortic endothelial cells

**DOI:** 10.1186/1475-2840-9-31

**Published:** 2010-07-27

**Authors:** Rining Tang, Qing Li, Linli Lv, Houyong Dai, Min Zheng, Kunling Ma, Bicheng Liu

**Affiliations:** 1 Institute of Nephrology, Zhong Da Hospital, Southeast University, Nanjing 210009, China

## Abstract

**Background:**

Substantial evidence suggests that high glucose (HG) causes endothelial cell damage; however, the potential mechanism therein has yet to be clarified. The aim of this study was to investigate the influence of HG on the endothelial-to-mesenchymal transition (EndMT) and its relevance to the activation of the renin-angiotensin system.

**Methods:**

Primary human aortic endothelial cells (HAECs) were divided into three groups: a normal glucose (NG) group, HG group, and irbesartan (1 μM)-treated (HG+irbesartan) group. The concentration of angiotensin II in the supernatant was detected by radioimmunoassay. Pathological changes were investigated using fluorescence microscopy and electron microscopy. Immunofluorescence staining was performed to detect the co-expression of CD31 and fibroblast markers, such as fibroblast-specific protein 1 (FSP1). The expressions of FSP1 and α-SMA were detected by RT-PCR and Western blot.

**Results:**

The treatment of HAECs in the HG group resulted in significant increases in the expressions of FSP1 and angiotensin II in dose-and time-dependent manners. The incubation of HAECs exposure to HG resulted in a fibroblast-like phenotype, wherein increased microfilamentation and a roughened endoplasmic reticulum structure were observed in the cytoplasm. The expressions of FSP1 and α-SMA were significantly increased in the HG group, and these changes were inhibited by irbesartan treatment (*P *< 0.05). Double staining of the HAECs indicated a co-localization of CD31 and FSP1 and that some cells acquired spindle-shaped morphologies and a loss of CD31 staining; however, treatment with irbesartan attenuated the expression of EndMT (*P *< 0.05).

**Conclusions:**

These findings suggest a novel mechanism in HG-induced endothelial damage via the mediation of the EndMT by angiotensin II, which was inhibited by Irbesartan.

## Background

Vascular complications, such as cardiomyopathy and nephropathy, are the leading cause of morbidity and mortality in patients with diabetes. Because the initial injury by hyperglycemia occurs in the blood vessels, endothelial cells are considered to be the first target, and, furthermore, endothelial damage plays an important role in the development and progression of diabetic vascular complications [1-3]. Four main molecular mechanisms have been implicated in glucose-mediated vascular disease: the glucose-induced activation of protein kinase C isoforms, an increased formation of glucose-derived advanced glycation end-products (AGEs), an increased glucose flux through the aldose reductase pathway, and an increased production of reactive oxygen species [4]; however, the mechanisms of endothelial injury by high glucose (HG) are not fully understood.

Recent studies have indicated that the endothelial-to-mesenchymal transition (EndMT) could contribute to the progression of diabetic nephropathy, diabetic renal fibrosis, and cardiac fibrosis [5-7], and that the rennin-angiotensin system (RAS) may be involved. Irbesartan is an angiotensin II (Ang II) receptor type 1 blocker (ARB) and has been shown to reduce vascular endothelial damage, improve hyperglycemia-induced endothelial dysfunction, and inhibit endothelial transdifferentiation into myofibroblasts in valve leaflets [8-11]. The aim of this study was to explore the influence of HG on the EndMT and its relevance in the activation of the RAS in HAECs.

## Materials and methods

### Cell culture

HAECs were purchased from Sciencell (No. 6100) and grown in a Sciencell endothelial basal medium (ECM, No. 1001). This ECM consists of 500 ml of basal medium, 25 ml of fetal bovine serum (No. 0025), 5 ml of endothelial cell growth supplement (No. 1052), and 5 ml of a penicillin/streptomycin solution (No. 0503).

Cells were cultured at 37°C in a humidified atmosphere with 5% CO_2_. The medium was changed every other day until the culture was approximately 50% confluent. When the culture reached 50% confluence, the medium was changed every day until the culture was approximately 80% confluent. HAECs were performed between the 2-4 passages. The culture medium was changed to a serum-free solution for 24 h, and the HAECs were treated with normal glucose (NG; 5.5 mM), HG (15 mM or 30 mM D-glucose) [12], or 5.5 mM NG + 24.5 mM mannitol for 48 h. These cells were exposed to HG (media that contained 5.5, 15, or 30 mM D-glucose) for 0, 6, 12, 24, 48, and 72 h. Some of the cells that were exposed to HG (30 mM) were also incubated with irbesartan (1 μM, Sanofi-aventis, France) [13] for 48 h.

### Ang II measurement

Ang II was measured in the supernatant by radioimmunoassay, as previously described [14]. A commercial radioimmunoassay kit (Beifang, China) was used for the Ang II measurement. On the basis of the time course of Ang II synthesis, HAECs were exposed to HG (30 mM) for 48 h.

### RT-PCR analysis

Total RNA was prepared from the HAECs using TRIzol (Key GEN). Total RNA was prepared using TRIzol (Key GEN) from HAEC. PCR reactions were performed using specific primer pairs: a FSP1 sense primer: 5' TTGGGGAAAAG GACAGATGAAG 3', anti-sense primer: 5'TGAAGGAGCCAGGGTGGAAAAA 3'), α-SMA sense prime: 5'ATAACATCAAGCCCAAATCTGC3', anti-sense primer: 5' TTCCTTTTTTCTTTCCCAACA 3') and a GADPH sense primer: 5'AAGGTCG GAGTCAACGGATTT 3', antisense primer: 5'AGATGATGACCCTTTTGGCTC 3').

### Western blot analysis

Equal amounts of cell lysate proteins (30 μg) were separated on 4-20% SDS-polyacrylamide gels and transferred onto nitrocellulose membranes (Pall, USA).

The membranes were incubated overnight with polyclonal rabbit anti-rat FSP1 and the polyclonal rabbit anti-rat α-SMA (Abcam, England), followed by a horseradish peroxidase-labeled goat anti-rabbit IgG (Key GEN, China). The signals were detected using an ECL advance system (GE Healthcare, UK).

### Immunofluorescent Staining

For a double immunofluorescence procedure, we incubated the HAECs with two primary antibodies at 4°C overnight. The primary antibodies were monoclonal mouse anti-CD31 (Santa Cruz Biotechnology, Europe) and polyclonal rabbit anti-FSP1 (Abcam, England). We incubated cells in 1% BSA for 1 h at room temperature in the dark with a mixture of two secondary antibodies and two different fluorochromes: Rhod red-conjugated goat anti-rabbit and FITC green-conjugated goat anti-mouse. As a negative control, the primary antibody was replaced with non-immune IgG, and no staining could be observed. FSP1^+ ^cells were observed to have oval and elongated shapes in the HG group. The pictures were captured by the LSM5 image browser (Zeiss) and analyzed using a laser scanning confocal microscope (LSM 510 META, Zeiss).

### Morphological analysis

Ultra-thin cells were counter-stained with uranyl acetate and lead citrate and were examined with a transmission electron microscope (HITACHI H600, TEM). The LSM5 image browser (Zeiss) was used to capture images of morphological changes in the HAECs using CD31 immunofluorescence staining, as previously mentioned.

### Statistical analysis

Data were expressed as mean ± standard deviation (SD) and analyzed by one-way analysis of the variance (ANOVA) using SPSS, version 13.0. Data were considered significant if *P *< 0.05.

## Results

### HG exposure dose response and time course on HAEC angiotensin II production

**Figure 1:**
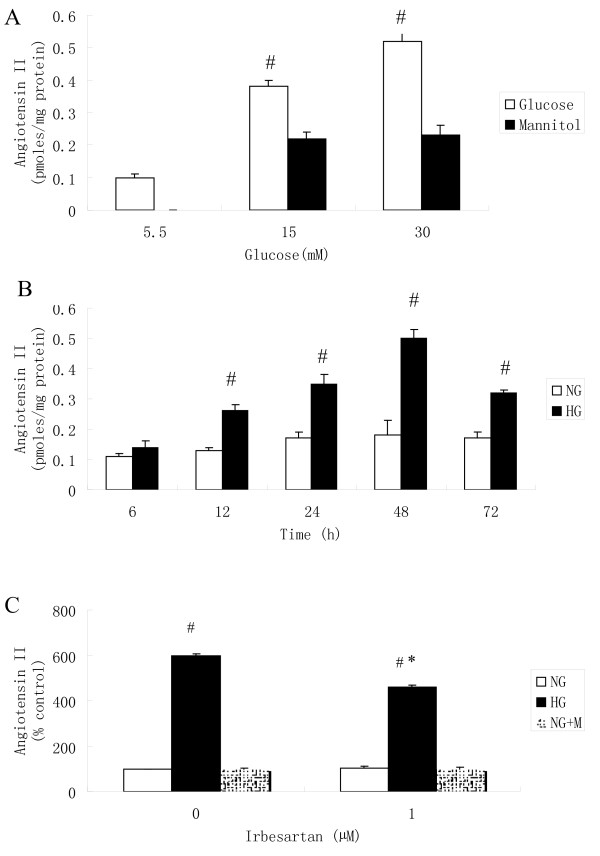
**The dose and time dependency of HG-stimulated Ang II synthesis in HAECs**. A: HAECs grown in a culture medium consisting of 5.5 mM, 15 mM, or 30 mM glucose for 48 h. Mannitol was used as a control for hyperosmolarity. B: HAECs grown in a 30 mM glucose medium for 6-72 h. Ang II was measured in the supernatant using radioimmunoassay. C: High glucose stimulated Ang II synthesis in the supernatant in the absence or presence of irbesartan in HAECs. HAECs that were grown in a serum-free normal glucose medium (NG, 5.5 mM) for 24 h and exposed to high glucose (HG, 30 mM) or in 5.5 mM glucose + 24.5 mM mannitol (NG+M) in the absence or presence of 1 μM irbesartan for 48 h. Irbesartan partially inhibited Ang II production in the culture medium. The depicted values are the means mean ± SD from three independent experiments that were performed in duplicate. # *P *< 0.05 vs. mannitol (A) or NG (B). *P < 0.05 vs. HG without inhibitor.

To demonstrate that enhanced Ang II production depended on the concentration and duration of HG exposure, we incubated HAECs in a medium that contained 5.5, 15, or 30 mM glucose for 48 h. Mannitol was added to the control cell incubation medium to equalize the osmolarity. Ang II was observed to increase in a dose-dependent manner in response to HG exposure (Fig. 1A). The concentration of Ang II in HG-exposed cells (30 mM) increased as early as 12 h and continued to increase until 48 h after exposure (Fig. 1B). As can be observed in Fig. 1C, irbesartan partially inhibited Ang II production in the culture medium.

### The Effect of irbesartan on the mRNA expression of FSP1 and α-SMA

**Figure 2:**
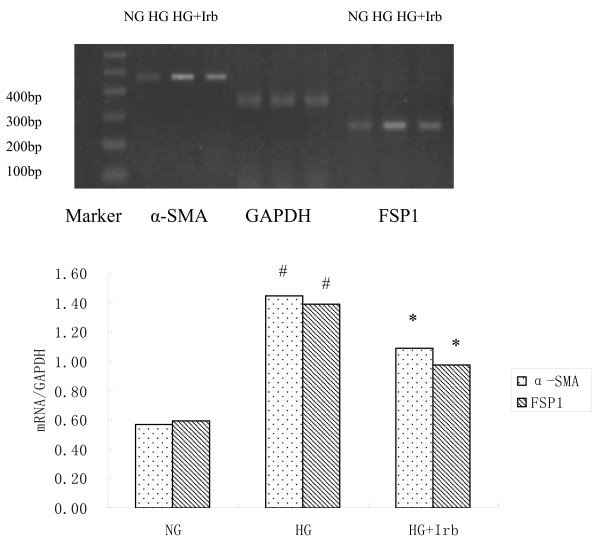
**The effect of irbesartan on the mRNA expression of FSP1 and α-SMA**. ^#^*P *< 0.05 vs. NG. and *P < 0.05 vs. HG. NG: normal glucose. HG: high glucose. HI+Irb: high glucose + Irbesartan.

As shown in Fig. 2, FSP1 and α-SMA mRNA expressions in HAECs exposure to HG were markedly up-regulated in comparison to NG group, which were inhibited by treatment with irbesartan (*P *< 0.05).

### The effect of irbesartan on the protein expression of FSP1 and α-SMA

**Figure 3:**
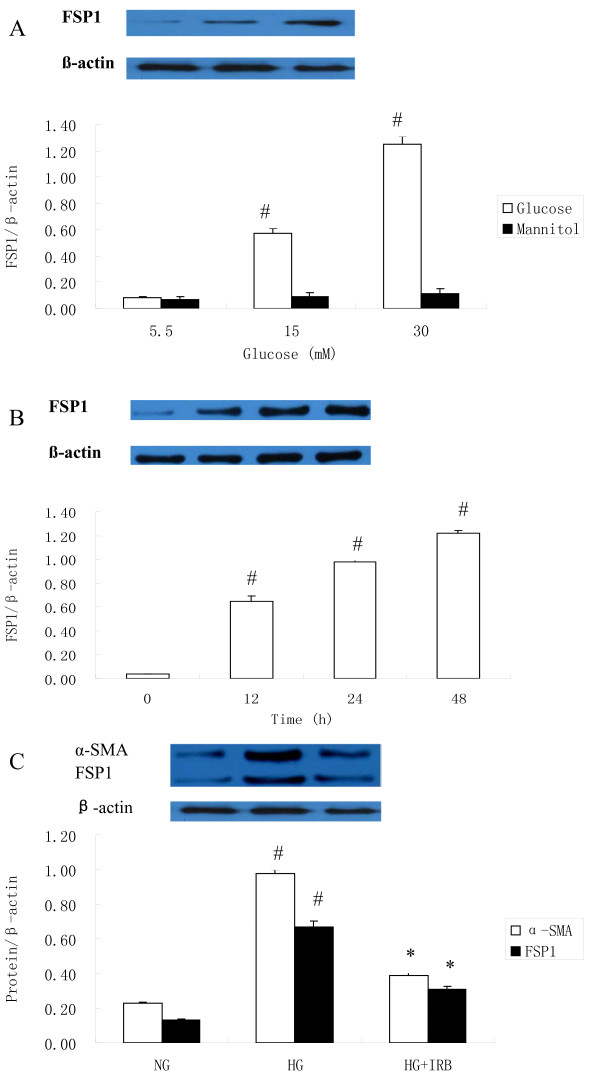
**Western blot analysis of the effect of HG on the synthesis of FSP1 and α-SMA protein in HAECs**. A: HAECs were grown in a culture medium that contained 5.5-30 mM glucose for 48 h. Mannitol was used as a control for hyperosmolarity. B: HAECs were incubated with HG (30 mM) for 0, 12, 24, and 48 h. (0 h: 0.04 ± 0.001, 12 h: 0.65 ± 0.04, 24 h: 0.98 ± 0.04, and 48 h: 1.22 ± 0.02; ^#^*P *< 0.05 vs. the control). C: Western blot analysis of the effect of HG on the synthesis of α-SMA and FSP1 protein in HAECs. Values are means ± SD; ^#^*P *< 0.01 vs. NG and * *P *< 0.05 vs. HG. NG: normal glucose. HG: high glucose. HI+Irb: high glucose + Irbesartan. Experiments were repeated three times. Irb: Irbesartan

According to Fig. 3A-B, after exposing a confluent monolayer of cells with HG at different concentrations and periods of time, it can be observed that after 48 h of exposure to increased HG concentrations, the FSP1 protein was progressively up-regulated (NG 5.5 mM: 0.08 ± 0.01, HG 15 mM: 0.57 ± 0.04, HG 30 mM: 1.25 ± 0.06; ^#^*P *< 0.05 vs. NG), reaching a peak at 30 mM HG with a 15.62-fold increase in comparison to that with NG exposure (Fig. 3A). In response to 30 mM HG, the introduction of HG time-dependently induced the synthesis of FSP1 protein (0 h: 0.04 ± 0.001, 12 h: 0.652 ± 0.04, 24 h: 0.98 ± 0.04, and 48 h: 1.22 ± 0.02; *P *< 0.05 vs. the control; Fig. 3B). Furthermore, FSP1 and α-SMA protein expression in HAECs exposure to HG were markedly up-regulated in comparison to NG group, which were inhibited by treatment with irbesartan (*P *< 0.05).

### Confocal microscopic analysis

**Figure 4:**
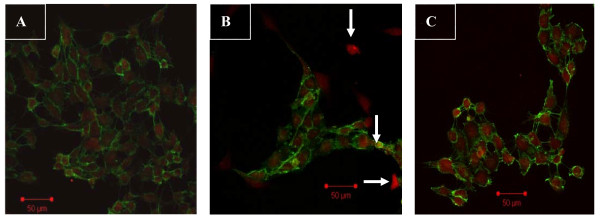
**Irbesartan inhibited the high glucose-induced EndMT in HAECs according to laser-scanning confocal microscopy**. Representative immunofluorescence staining of CD31 (green) and FSP1 (red) were observed. A merging of both images reveals populations of cells acquired FSP1 expression and lost CD31 expression (arrows, B). The administration of irbesartan reduced the number of co-localization of CD31 and FSP1 (C, *P *< 0.05). A: normal glucose as controls; B: Treated with HG (30 mM) for 48 h. C: Treated with HG (30 mM) + irbesartan (1 μM) for 48 h. Experiments were repeated three times. NG: normal glucose. HG: high glucose. HI+Irb: high glucose + Irbesartan.

We performed labeling experiments using antibodies to CD31 (endothelial cell marker; green) and fibroblast markers FSP1 (red, also termed S100A4). Confocal microscopy revealed the co-localization of both FSP1 and CD31 (Fig. 4B). An analysis of FSP1/CD31 double labeling revealed that some cells acquired FSP1 staining and lost CD31 staining, which suggests that the EndMT occurred. The administration of irbesartan markedly reduced the number of such double-staining cells (Fig. 4C, *P *< 0.05). In the control cells, FSP1 expression was confined to sparsely scattered fibroblasts (Fig. 4A).

### Morphological analysis

**Figure 5:**
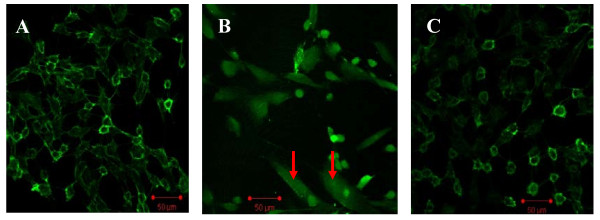
**Immunofluorescence staining of HAECs with CD31 in various groups**. The Incubation of HAECs with high glucose (30 mM) for 48 h resulted in a fibroblast-like phenotype (B). Treatment with irbesartan could significantly prevent the morphological changes (C). NG: normal glucose. HG: high glucose. HI+Irb: high glucose + Irbesartan.

Normal endothelial monolayers displayed a typical cobblestone morphology. We observed that HAECs exposure to HG for 48 h exhibited profound changes with cells becoming elongated, spindle-shaped and lost cobblestone morphologies according to fluorescence microscopic analysis (Fig. 5B). Interestingly, treatment with irbesartan was observed to significantly prevent these morphological changes (Fig. 5C).

**Figure 6:**
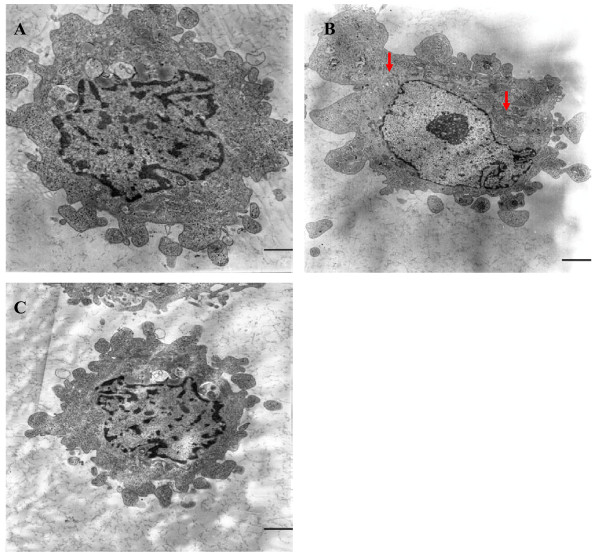
**Cellular ultrastructure following HG treatment**. Transmission electron microscopy depicts the change in cellular ultrastructure following HG (30 mM) exposure (magnification × 6,000). It can be seen that normal HAECs present with few microfilaments and a rough endoplasmic reticulum (A). After exposure to HG, microfilamentation and a swollen rough endoplasmic reticulum appeared in the cytoplasm (B). These changes were attenuated by treatment with irbesartan (C). 1 bar = 4 μm.

Electron microscopy analysis of the control demonstrated that the endothelial cells therein exhibited normal structures (Fig. 6A, × 6000). In contrast, the HG group that was treated with HG (30 mM) for 48 h exhibited endothelial protrusion, a significantly roughened endoplasmic reticulum, and microfilamentation (arrows, Fig. 6B, × 6000). These changes were attenuated by treatment with irbesartan (Fig. 6C, × 6000).

## Discussion

Microangiopathy is the most common complication in diabetes, wherein endothelial cell injury is an early feature of microvascular lesions. Studies have shown that endothelial injury accelerates atherosclerosis and subsequently causes cardiovascular events [15]. Emerging evidence has shown that hyperglycemia may have a direct role in endothelial cell injury [16-18], which is characterized by cell apoptosis. More recently, it has been shown that the endothelium may develop the EndMT, which has been found to be involved in cardiac fibrosis and tubulointerstitial fibrosis in animal models [6,7,19,20]; however, the potential mechanisms therein are still largely assumptive. In this study, we found that when HAECs were exposed to HG, they developed a series of phenotypical changes, such as a spindle-shaped morphology, an increasingly roughened endoplasmic reticulum, and microfilamentation. Moreover, these cells expressed FSP1 and α-SMA, which suggests the occurrence of the EndMT.

Although the EndMT was first investigated as a critical process in heart development [21], it is now clear that the EndMT can also postnatally occur in various pathological settings, including cardiac fibrosis, renal fibrosis, and diabetic nephropathy. Recent studies have shown that the EndMT also contributes to the development of diabetic renal interstitial fibrosis, diabetic nephropathy, and cardiac fibrosis [5,7,19], which indicate a relationship between the EndMT and fibrosis. Cardiac and renal fibroses are also the most common diabetic vascular complications [22-24]. Chronic hyperglycemia is a major initiator of diabetic vascular complications. Indeed, HG via various mechanisms, such as an increased production of oxidative stress, AGEs, and the activation of the RAS and protein kinase C [4,25], promotes cardiac and renal fibroses. Therefore, whether HG directly induces the EndMT in HAECs is an interesting question that has not been previously addressed. In this study, our findings demonstrate that double-stained HAECs exposure to high glucose exhibited the co-localization of CD31 and FSP1, and some cells acquired spindle-shaped morphologies and a loss of CD31 staining. Furthermore, the expressions of FSP1 and α-SMA were significantly increased in the HG group, which strongly indicates an HG-induced EndMT and could be an important mechanism in diabetic vascular complications.

How did HG induce EndMT? In our study, we observed that irbesartan as an ARB significantly inhibited the EndMT. Furthermore, other studies have demonstrated the antiproteinuric effects and the preservation of endothelial function that derive from ARB, which translate into cardiovascular and renoprotective benefits that extend beyond the lowering of blood pressure [26,27]. In vitro and in vivo studies have found that irbesartan could ameliorate endothelial function in hypertension and diabetes, which are two frequent diseases where endothelium homeostasis alterations are typically present [27]. In addition, irbesartan therapy has been demonstrated to improve metabolic risk factors in clinical settings [28,29]; however, the exact mechanisms of the cardiovascular and renoprotective benefits that derive from irbesartan therapy are not fully understood. In this study, we found that HG directly stimulated angiotensin II synthesis in HAECs, and irbesartan markedly protected endothelial cells from HG-induced injury. Because the EndMT may be an early event in the pathogenesis of fibrosis [19,30], our findings suggest that early treatment with ARB might be an important strategy in the prevention of microvascular disease that is complicated by diabetes.

## Conclusions

These findings suggest a novel and early mechanism concerning HG-induced endothelial damage via an angiotensin II-mediated EndMT, which provides new insight into the early application of ARB in the protection of blood vessels and the prevention of organ failure in diabetes.

## Abbreviations

EndMT: endothelial-to-mesenchymal transition; HAECs: human aortic endothelial cells; HG: high glucose; Irb: Irbesartan; FSP1: fibroblast-specific protein1; AGEs: advanced glycation end-products; RAS: rennin-angiotensin system; Ang II: angiotensin II; ARB: angiotensin II receptor type 1 blocker.

## Competing interests

The authors declare that they have no competing interests.

## Authors' contributions

TR performed the experiments, analyzed data, interpreted results, and wrote the manuscript. LQ participated in the HAEC culture and analysis. LL and DH carried out the RT-PCR and Western blotting. ZM helped to carry out the immunofluorescent staining. MK coordinated the study and was involved in the data interpretation. LB participated in the study design and coordination and helped review the manuscript. All authors read and approved the final manuscript.
